# Critical Analysis of the Melanogenic Pathway in Insects and Higher Animals

**DOI:** 10.3390/ijms17101753

**Published:** 2016-10-20

**Authors:** Manickam Sugumaran, Hanine Barek

**Affiliations:** Department of Biology, University of Massachusetts Boston, Boston, MA 02125, USA; Haning.barek001@umb.edu

**Keywords:** melanogenesis, sclerotization, tyrosinase, phenoloxidase, laccase, dopachrome tautomerase, eumelanin, dopamine, dopa

## Abstract

Animals synthesize melanin pigments for the coloration of their skin and use it for their protection from harmful solar radiation. Insects use melanins even more ingeniously than mammals and employ them for exoskeletal pigmentation, cuticular hardening, wound healing and innate immune responses. In this review, we discuss the biochemistry of melanogenesis process occurring in higher animals and insects. A special attention is given to number of aspects that are not previously brought to light: (1) the molecular mechanism of dopachrome conversion that leads to the production of two different dihydroxyindoles; (2) the role of catecholamine derivatives other than dopa in melanin production in animals; (3) the critical parts played by various biosynthetic enzymes associated with insect melanogenesis; and (4) the presence of a number of important gaps in both melanogenic and sclerotinogenic pathways. Additionally, importance of the melanogenic process in insect physiology especially in the sclerotization of their exoskeleton, wound healing reactions and innate immune responses is highlighted. The comparative biochemistry of melanization with sclerotization is also discussed.

## 1. Introduction

Melanins are a group of widely distributed phenolic biopolymers arising from the amino acid, tyrosine and related phenolic compounds [1,2,3]. They are responsible for the skin, hair (or coat or feather), and eye color in animals. Plants as well as fungi and even some bacteria synthesize melanin not only from tyrosine but also from a variety of different phenolic compounds. Animals synthesize three different kinds of melanin viz., pheomelanin, eumelanin and neuromelanin by the action of ubiquitously present enzyme tyrosinase as well as phenoloxidases. The yellow to red pigment found in the red hair, feather and fur of animals is due to pheomelanin. Pheomelanin is produced by the oxidative polymerization of cysteinyldopa, which in turn is synthesized by the coupling of enzymatically generated dopaquinone with the amino acid, cysteine. The brown to black eumelanins are formed by the oxidative polymerization of dihydroxyindoles that are derived from dopa and its derivatives. Neuromelanin is produced in substantia nigra and is made up of dopamine derived polymeric materials. Studies on the biosynthesis, properties, function and structure of melanins have been thoroughly examined by numerous research groups worldwide for several decades [1,2,3,4,5,6,7,8,9]. However, several aspects of melanin biochemistry including biosynthesis, metabolism, structure, biological and molecular biological aspects of the enzymes associated with the process are yet to be unraveled. The focus of this article will be limited to the biosynthesis, some functions and comparative biochemistry of melanogenesis and related processes in insects and higher animals.

## 2. Raper–Mason Pathway for the Biosynthesis of Eumelanin

**Figure 1:**
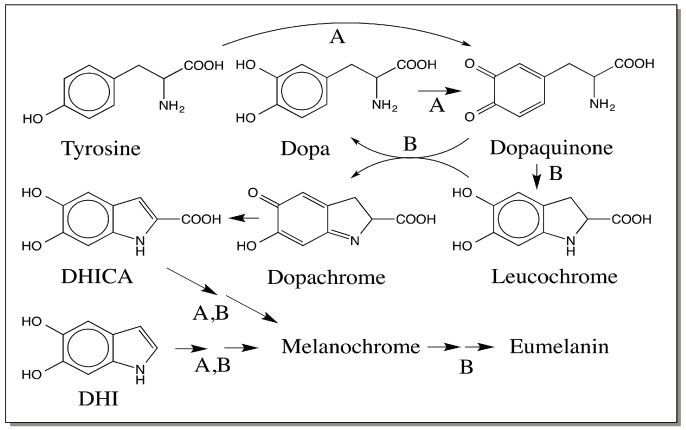
Raper–Mason pathway for the biosynthesis of melanin. The bifunctional enzyme, tyrosinase (A) converts tyrosine and dopa to dopaquinone. Dopaquinone undergoes instantaneous intramolecular nonenzymatic cyclization forming leucochrome, which is rapidly oxidized by dopaquinone to dopachrome. The red colored dopachrome is converted to 5,6-dihydroxyindole (DHI) as the major product and 5,6-dihydroxyindole-2-carboxylic acid (DHICA) as the minor product. Oxidative polymerization of dihydroxyindoles produces the melanin pigment. Tyrosinase is assumed to be the sole enzyme associated with this pathway and the rest of the reactions (B) are presumed to be of nonenzymatic origin.

Classical studies carried out on melanin biosynthesis about a century ago by Raper and his associates established the intermediacy of dopa, 5,6-dihydroxyindole (DHI) and 5,6-dihydroxyindole-2-carboxylic acid (DHICA) in eumelanin biosynthesis [10,11,12]. Using partially purified enzyme preparations from the mealworm *Tenebrio molitor*, Raper oxidized tyrosine to the red colored (dopachrome) stage and allowed the reaction to undergo further transformation. From this reaction mixture, he isolated and characterized DHI as the major product, and DHICA as the minor product. Therefore, he deduced that the oxidative polymerization of DHI along with minor amount of DHICA polymerization leads to the production of insoluble melanin pigment [10,11,12]. Later Mason identified dopachrome as the red pigment and confirmed its conversion to DHI [13,14]. He also demonstrated the two-electron oxidation of catechols to quinones. Thus, the successful identification of dopaquinone, dopachrome, DHI and DHICA as an important intermediates of eumelanogenesis, established by the pioneering work of Raper and Mason led to the proposal shown in Figure 1 for the biosynthesis of melanin. This simplistic view was accepted widely. Even though the proposal acknowledges the minor role of DHICA, it assumes that DHI is the major precursor for melanin in animals. After dopaquinone formation, the rest of the reactions in this pathway could proceed nonenzymatically and spontaneously without the need for any enzyme resulting in dark colored insoluble melanin pigment in test tubes. Therefore, for a long time, the common understanding is that tyrosinase is the sole enzyme responsible for melanin biosynthesis.

## 3. Discovery of Dopachrome Tautomerase

The popular belief that tyrosinase is the only enzyme associated with melanogenesis was shattered with the discovery of dopachrome converting enzyme in 1980s [15,16]. Pawelek and associates discovered a new enzyme in mammalian system that bleached the red color of dopachrome and called this enzyme as dopachrome-converting enzyme or dopachrome conversion factor. The product of the reaction was initially identified as DHI. Purification and characterization of this new enzyme proved to be extremely difficult as it is always found to be associated with tyrosinase [17]. Subsequently, various group of workers characterized the enzyme devoid of tyrosinase and demonstrated that the reaction catalyzed by this enzyme is dopachrome conversion to DHICA [18,19,20]. Therefore, this enzyme is named as dopachrome isomerase [18] or dopachrome tautomerase [19,20]. However, the most widely accepted and currently used name for this enzyme is dopachrome tautomerase (DCT) [19,20]. In insects [21,22,23] and cuttlefish [24], a different enzyme that catalyzes the conversion of dopachrome to DHI was characterized.

## 4. Insect Dopachrome Decarboxylating Enzyme Is Also a Tautomerase

**Figure 2:**
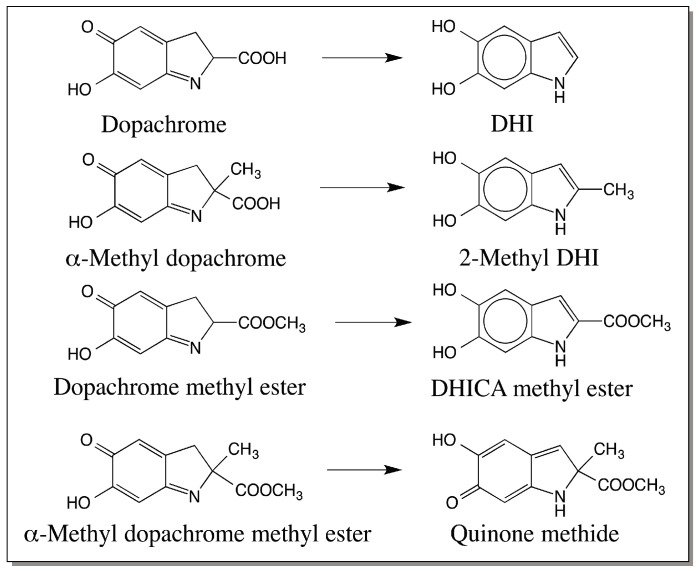
Reactions catalyzed by insect dopachrome conversion factor. Insect dopachrome conversion factor catalyzes decarboxylative rearrangement of dopachrome to DHI. It also accepts α-methyl dopachrome as the substrate and produces 2-methyl DHI as the sole product. If dopachrome methyl ester is provided as the substrate, it uniquely converts it to DHICA methyl ester, thus acting as a typical tautomerase. Finally with α-methyl dopachrome methyl ester, which can neither undergo decarboxylation nor deprotonation at α-carbon atom, the enzyme causes an isomerization producing the stable quinone methide.

As stated above, insect dopachrome conversion enzyme produces DHI as the product [21,22,23]. Therefore, many scientists wondered if this enzyme should also be called dopachrome tautomerase. However, a careful examination of the substrate specificity of the enzyme revealed that the insect dopachrome conversion factor is indeed a tautomerase [23]. Insect enzyme exhibits wide substrate specificity and attacks a number of l-dopachrome derivatives, but not d-dopachromes [23]. If l-dopachrome is provided as the substrate, it readily decarboxylates the substrate and produces DHI. Similarly if α-methyl dopachrome is provided, it generates 2-methyl DHI (Figure 2). Therefore, the insect enzyme appears to be a decarboxylase. However, surprisingly, it also attacks dopachrome methyl ester producing its isomeric product, DHICA methyl ester (Figure 2). In fact, this compound serves as a better substrate than dopachrome or α-methyldopachrome for the insect enzyme. In this instance, it behaves very much like the mammalian enzyme in catalyzing the simple isomerization reaction. What is even more interesting is its ability to attack α-methyldopachrome methyl ester, which can neither undergo deprotonation at the α-carbon atom nor decarboxylation. However, this compound is converted into a stable quinone methide and is discharged from the active site as the sole product (Figure 2). These studies not only established the transient formation of quinone methide as a reactive intermediate during melanin biosynthesis, but also confirmed that the insect enzyme is indeed an isomerase [23]. Thus, the insect enzyme catalyzes the decarboxylation of some dopachrome derivatives and tautomerization of others. To distinguish it from the mammalian DCT, this enzyme can be designated as dopachrome decarboxylase/tautomerase (DCDT).

## 5. Mechanism of Dopachrome Conversion Reaction

**Figure 3:**
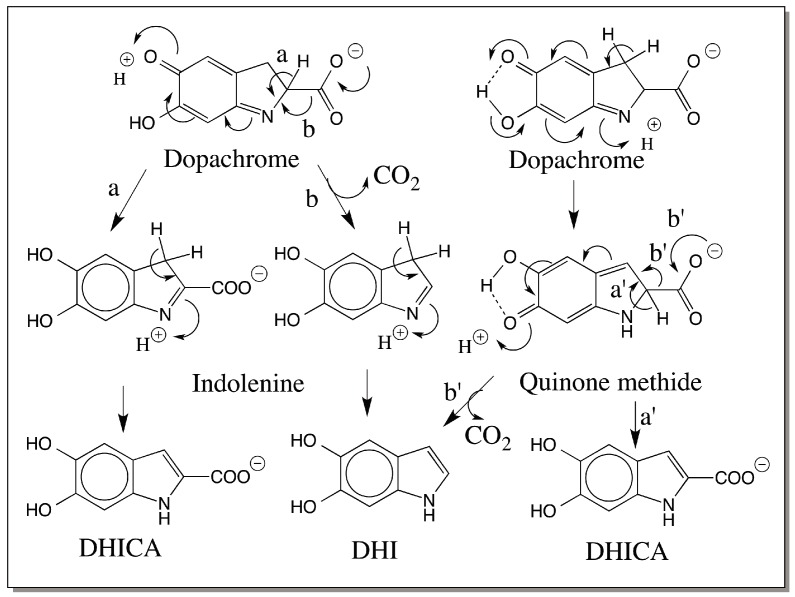
Two possible routes for the conversion of dopachrome. Dopachrome can undergo either (a) deprotonation or (b) decarboxylation to generate two different indolenine derivatives. Rapid aromatization of these unstable intermediates will generate DHICA and DHI respectively. However, this route is unlikely to operate. On the other hand, a common quinone methide intermediate formed by isomerization of dopachrome can either lose proton (route a′) or carboxyl group (route b′) depending upon the reaction conditions producing DHI or DHICA.

Earlier studies advocated the intermediacy of an indolenine derivative for dopachrome conversion reaction (Figure 3) [25]. However the indolenine route assumes electron donating properties for the C=N in dopachrome. In reality it is electron withdrawing in the direction of C=N bond. Indeed, it is this property that makes dopachrome stable as the two groups C=O and C=N exhibit opposite polarity and stabilize the dopachrome structure. This route also generates two different indolenine derivatives—one that retains the carboxyl group and the other that has undergone decarboxylation (Figure 3). On the other hand, our group first showed that quinone methide is the transient intermediate formed during melanogenesis both in chemical reaction and in enzymatic reactions [23,26] by demonstrating the facile isomerization of α-methyl dopachrome methyl ester to a stable quinone methide that can be isolated and characterized [26]. Subsequently, Prota and his associates also confirmed the quinone methide formation both by characterizing the quinone methide produced from α-methyl dopachrome methyl ester and by demonstrating primary kinetic isotope effect with the 3,3-dideuterated dopachrome [27,28]. As shown in Figure 3, the decarboxylation will be the rate-determining step for DHI production from indolenine route. Hence substitution of deuterium at 3-position should have no effect on rate of conversion of dopachrome to DHI. However, a large kinetic isotope effect is observed for this reaction thereby discounting the indolenine route [27]. For DHICA production also, indolenine route will be less favorable than the quinone methide route because the rate determining step would be removal of proton from the 2-position and not from 3-position.

A single quinone methide intermediate can account for both DHI production and DHICA production (Figure 3). This route will also acknowledge the large primary kinetic isotope effect observed between the protium form and the deuterium form of the 3-substituted dopachrome [27]. Quantum chemical calculations of copper chelated dopachrome also supports this contention and confirms that deprotonation of the β-proton is energetically most favorable leading to quinone methide production [29]. Once formed, quinone methide can undergo two different reactions. Decarboxylation coupled aromatization of the quinonoid ring will produce DHI as the product. Decarboxylation of quinone methide is naturally a facile reaction as it is the β,γ-unsaturated carboxy compound. Nondecarboxylative isomerization will generate DHICA as the product. Both reactions seem to occur in nature. Even with the enzyme-catalyzed reaction, mammalian DCT takes up the nondecarboxylative tautomerization route producing DHICA as the product [18,19,20], and the insect DCDT produces DHI as the product [21,22,23]. With nonenzymatic reaction, depending on the presence or absence of bivalent metal ions the ratio of DHICA to DHI seems to vary [25]. In chemical conversions of dopachrome carried out at pH 6.5, DHI is produced as the major product. When the pH was changed to 13, DHICA is produced as the major product under the same conditions thereby showing the influence of pH on the rearrangement of dopachrome [30].

## 6. Dopachrome Conversion—5,6-Dihydroxyindole (DHI) versus 5,6-Dihydroxyindole-2-carboxylic Acid (DHICA)

To best of our knowledge, all known insect dopachrome DCDTs convert dopachrome to DHI and all mammalian DCTs produce DHICA as the product. Why is there such a marked difference exhibited by these two enzymes which act on the same substrate? Different explanations are possible to account for this difference. The first one is related to the metal ion present at the active site. The mammalian DCT is a metalloprotein having zinc at its active site [31,32]. Therefore, it is possible that this enzyme mimics the nonenzymatic bivalent metal ion assisted dopachrome conversion producing DHICA as the product [25]. The nature of the metal ion present at the active site of insect DCDT is yet to be determined. If metal ions were absent at the active site, insect enzyme reaction would mirror the nonenzymatic conversion of dopachrome to DHI [25]. Insect enzyme might eject quinone methide intermediate from the active site as observed in the case of α-methyl dopachrome methyl ester [26]. The ejected quinone methide being a β,γ-unsaturated acid, will undergo spontaneous decarboxylation, generating DHI (Figure 3). If the metal ion present in insect enzyme is also zinc, then alternate explanations have to be considered.

**Figure 4:**
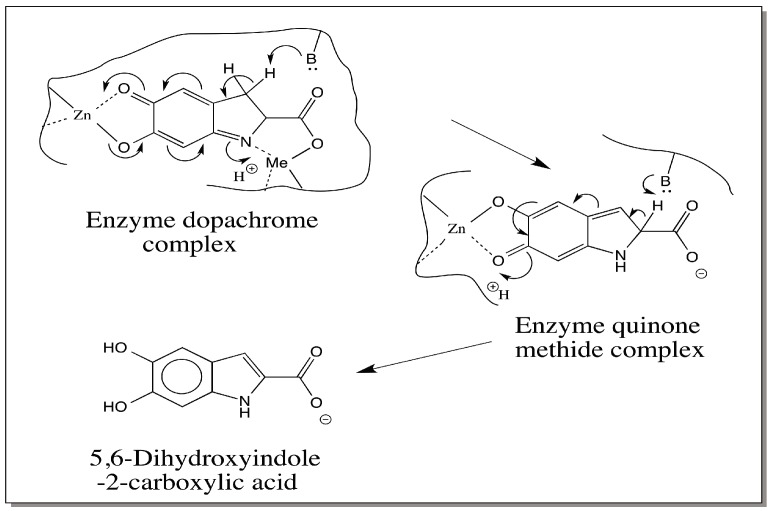
One possible mechanism for dopachrome conversion to DHICA by mammalian dopachrome tautomerase (DCT). Mammalian DCT possesses two metal binding sites. If one metal ion binding site is occupied by the quinonoid nucleus and the other by the amino group and carboxyl group, it would prevent the spontaneous decarboxylation of quinone methide intermediate thereby causing the isomerization to DHICA.

An interesting aspect about the nature of metal binding site present on mammalian DCT is its remarkable similarity to the copper-binding site of tyrosinase [32]. While binuclear copper is essential for oxygen activation, no such binuclear center is needed for dopachrome isomerization. Therefore, it is reasonable to assume that one zinc atom is bound to the quinonoid side and the other to the imine group and the carboxyl group as shown in Figure 4. This might stabilize the intermediate quinone methide and will allow its rearrangement to DHICA. The insect enzymes do not show any sequence homology to mammalian counterpart or even to insect phenoloxidases. Bioinformatics analysis did not reveal any kind of homology even at the metal binding site between the two classes of dopachrome converting enzymes. Insect DCDTs may not have the same kind of two metal binding sites on them. They may have one metal ion (or none) and might allow the quinone methide to leave the active site. The released quinone methide then undergoes nonenzymatic decarboxylative transformation producing DHI in this case.

**Figure 5:**
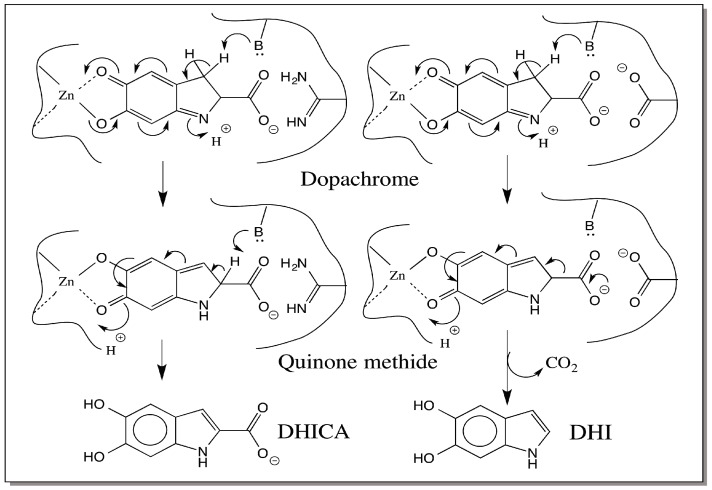
Possible role of active site in diverting the course of dopachrome conversion reaction. The crucial arginine reside at the metal binding site identified in the case of mammalian DCT might bind to the carboxyl group and protect it from decarboxylation reaction, thus allowing the production of the sole product, DHICA. Insect dopachrome decarboxylase/tautomerase (DCDT) may not have such a protective mechanism and might even have a carboxyl group that will favor the decarboxylation of quinone methide to DHI.

Finally, the amino acid residues present at the active site could also cause the differences in the course of the reaction. For instance, if the carboxyl group of dopachrome is situated close to the amino group of lysine or imidazole group of histidine, or guanido group of arginine present at the active site of the enzyme, it will be protected from decarboxylation by strong ionic interactions. The guanido group of arginine has the ideal geometry that is suitable for binding the carboxyl group. This will stabilize the quinone methide intermediate and favor the isomerization reaction leading to DHICA production in the case of mammalian DCT as shown in Figure 5. The *slaty* mutant that is defective in the activity of dopachrome isomerase has just one amino acid modification. Arginine in this protein is modified to glutamine (Arg^194^ → Gln), which is solely responsible for the drastic reduction in the activity of the enzyme [33,34]. This strongly suggests the critical role played by the arginine residue in catalysis and/or binding of substrate. Lack of such a group or presence of a carboxyl group could cause the ejection of quinone methide from the active site after dopachrome isomerization and subsequent nonenzymatic decarboxylation in the case of insect DCDT (Figure 5). Unfortunately, not much information is available on the structure of the insect enzyme to assess the role of amino acid side chain in altering the course of the reaction. Nevertheless, it is imperative that the active site geometry has a decisive role in diverting the reaction either to DHICA or in favor of DHI. Further studies are desperately needed to resolve this aspect.

## 7. DHICA Oxidase

Mammalian melanogenesis seems to be aided by at least three different proteins. They are tyrosinase, tyrosine-related protein 2, which is dopachrome tautomerase, and tyrosinase-related protein 1 or DHICA oxidase. The genes designated as *albino*, *slaty*, and *brown*, respectively, code for these three proteins in mouse [33,34,35,36,37]. DHICA turned out to be a poor substrate for mouse tyrosinase and hence, a successful search lead to the discovery of DHICA oxidase of brown loci as the third enzyme in melanogenic pathway [36,37]. In humans, however, tyrosinase seems to exhibit broader substrate specificity and is able to oxidize DHICA [38]. A 100-kDa membrane protein called Pmel 17/silver was also identified from human cell lines that is able to catalyze the conversion of DHICA to melanin [39]. DHICA oxidase is able to form heterodimeric structure with tyrosinase [40]. The metal ion present at the active site of this enzyme needs to be identified. More work is needed to shed light on the structure, function and mechanism of DHICA oxidase.

## 8. Modified Raper–Mason Pathway for the Biosynthesis of Eumelanin

**Figure 6:**
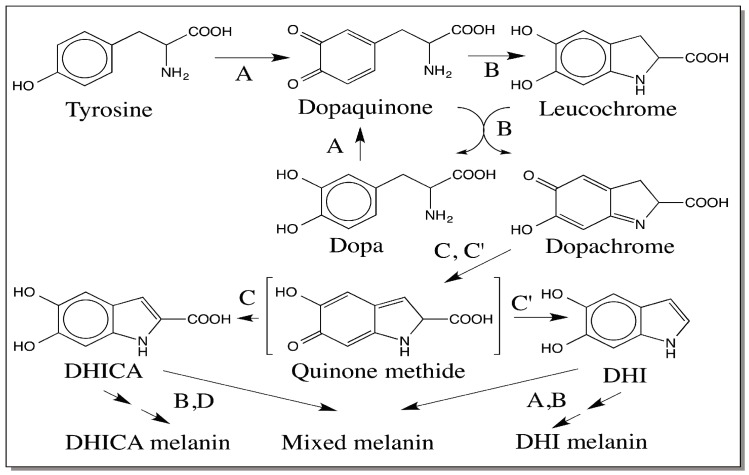
Modified Raper–Mason Pathway for the Biosynthesis of Eumelanin. Tyrosinase (A) catalyzes the oxidation of tyrosine to dopaquinone and conversion of dopa to dopaquinone. Dopaquinone undergoes intermolecular cyclization producing leucochrome, which undergoes double decomposition with dopaquinone generating dopa and dopachrome. Dopachrome is isomerized by DCT to the transient quinone methide intermediate that is converted to DHICA in mammals. Insect DCDT on the other hand produces DHI. Nonenzymatic transformations may also be responsible for DHI versus DHICA production in different systems. Oxidative polymerization of dihydroxyindoles generates different kinds of eumelanin pigments. (B = nonenzymatic reactions; C = mammalian DCT; C′ = insect DCDT; and D = DHICA oxidase).

Eventually all these discoveries led to the modification of the Raper–Mason pathway as shown in Figure 6. A note on the tyrosinase reaction should be mentioned here. Detailed mechanistic studies revealed that tyrosinase possess two different activities. The first activity is responsible for the conversion of tyrosine to dopaquinone directly [41]. The second activity is the typical *o*-diphenoloxidase activity that causes the oxidation of catechols to *o*-quinones. Thus tyrosinase converts tyrosine as well dopa to dopaquinone. Intramolecular cyclization of the dopaquinone leads to the generation of colorless leucochrome, which is further oxidized by dopaquinone to dopachrome that accumulates in the reaction mixture. During this process, dopaquinone itself gets reduced to dopa, which is further oxidized by tyrosinase to dopaquinone. Mammalian DCT converts dopachrome to DHICA and insect DCDT converts dopachrome to DHI via a common quinone methide intermediate. Dihydroxyindoles are oxidized to indolequinone, quinone methide and iminochrome isomers that will then polymerize producing DHI melanin, DHICA melanin and mixed DHI-DHICA melanin. The generation and subsequent reactions of semiquinones will also lead to the melanin production and account for the free radical nature of melanin polymer.

The bulk of the studies carried out in relation to Raper–Mason pathway indicate that DHI is the major product of dopachrome conversion while DHICA is a minor product [10,11,12,13,14]. However, mammalian DCT produces only DHICA and not DHI [18,19,20,33,34,35]. Moreover, DCT of mammalian origin seems to be associated with the tyrosinase as high molecular weight complex of tyrosinase and related proteins [17]. Such a metabolon complex will channel the product of one reaction into the active site of the other enzyme to ensure an efficient metabolic transformation. Complex formation also avoids the leakage of harmful quinonoid reactive intermediates. This constraint dictates the sole production of DHICA melanin and not DHI melanin. However, all three different eumelanins, viz., DHI melanin, DHICA-DHI mixed melanin and DHICA melanin, are formed in mammalian systems [3,5,42,43]. For this to occur, obviously DHI has to be synthesized some how in comparable quantities to that of DHICA or even more. It is possible that there is an undiscovered dopachrome-converting enzyme that specifically converts dopachrome to DHI in mammals, or nonenzymatic reactions are playing a crucial role to generate DHI in different melanogenic systems. It should be recalled here that various divalent metal ions are assisting the production of only DHICA in nonenzymatic reactions [25]. Thus the conditions for DHI production in melanogenic system have to be devoid of bivalent metal ions to ensure nonenzymatic decarboxylative transformation of dopachrome to DHI. Certainly more studies are needed to clarify the biosynthetic origin of DHI in mammalian systems. Similarly, in insects system the presence or absence of DHICA melanin should be unequivocally assessed.

## 9. Melanin from Other Catecholamines

**Figure 7:**
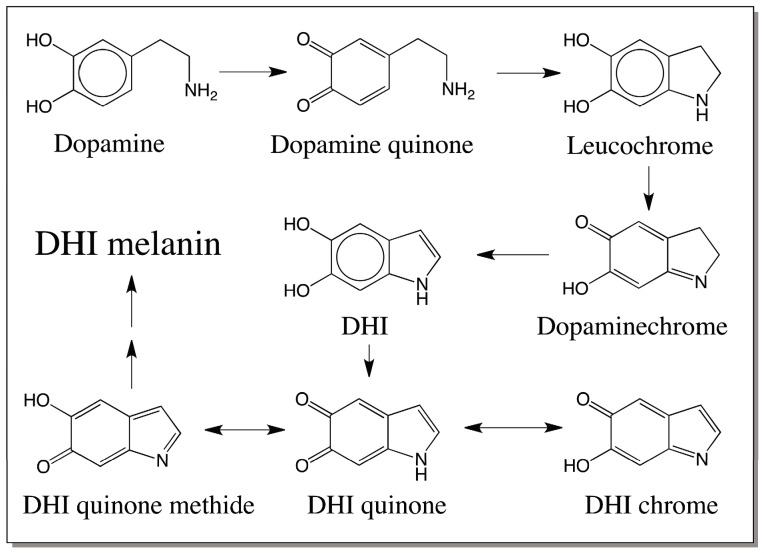
Melanin biosynthesis from dopamine. Tyrosinase or other oxidative enzymes/reactants will convert dopamine to dopaminequinone. Intramolecular cyclization to leucochrome and further oxidation of the leucochrome produces the dopaminechrome. Dopaminechrome is converted to DHI by isomerization reaction. Further oxidation of DHI to its quinonoid products and their eventual polymerization leads to dopamine melanins, which is DHI melanin.

Tyrosinase as well as phenoloxidases exhibit wide substrate specificity and oxidize a number of catecholic compounds. It is expected that not only dopa but also a variety of endogenous catecholamines that can serve as substrates for the oxidative enzymes, will participate in melanin biosynthesis. Oxidative enzymes also attack dopamine, a very common metabolite of dopa formed by the action of dopa decarboxylase, to dopaminequinone. Dopaminequinone exhibits the same kind of intramolecular cyclization and oxidation producing dopaminechrome. Conversion of dopaminechrome to DHI and its further oxidation will result in DHI melanin [44,45]. The course of the reactions leading to the generation of dopamine melanin is depicted in Figure 7. As is evident the pathway is very similar to the one proposed for dopa melanin with less simple reactions due to the lack of carboxyl group. What is intriguing in this pathway is the fact that dopaminechrome isomerization can proceed without the need for an enzyme [44,45], although some of the insect DCDTs have been shown to catalyze the conversion of dopaminechrome to DHI [46]. Dopamine-melanin seems to be the dominant form of neuromelanin in substantia nigra [47]. It is likely that dopamine and other catecholamines found in this tissue are readily oxidized either by metal ions or by other enzymes to reactive quinonoid species, which eventually polymerize to black colored neuromelanin [47]. Insects also seem to produce dopamine derived melanin much more than dopa derived melanin (see Section 11, Section 12 and Section 13). DHI can also react with molecular oxygen and undergo semiquinone formation, which will result in radical coupling reactions leading to DHI melanin. The structure as well as mechanism of formation of neuromelanin is still under investigation in several laboratories.

**Figure 8:**
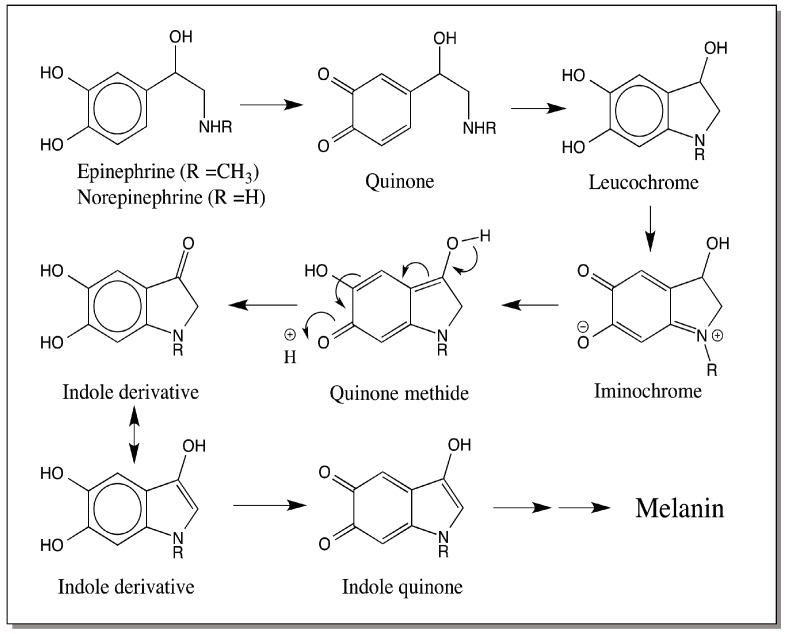
Melanin biosynthesis from epinephrine and norepinephrine. Both epinephrine and norepinephrine can be easily oxidized to their two-electron oxidation product—quinones. These quinones also exhibit rapid intramolecular cyclization producing leucochromes. Conversion of leucochrome to iminochrome and isomerization to quinone methide will result in adrenolutin formation in the case of epinephrine. A similar reaction of norepinephrine to the keto indole derivative has not yet been demonstrated, but is quite possible. Further oxidative transformations of these products will result in melanin production.

Norepinephrine (also known as noradrenaline) and its methylated derivative, epinephrine (also called adrenaline) can also form melanin pigment. They arise by the action of dopamine-β-hydroxylase, which converts dopamine to norepinephrine. Norepinephrine is subjected to methylation by a *N*-methyl transferase that adds a methyl group on to amine generating the catecholamine hormone—epinephrine. Both these catecholamines can be oxidized by tyrosinase and related oxidases to their corresponding quinones. These quinones will also exhibit intermolecular cyclization reaction much like dopaquinone and dopaminequinone producing leucochromes. Further oxidation of leucochrome and conversion to the transient quinone methide tautomer leads to a reaction that generates a keto catechol [48,49]. In the case of adrenaline oxidation, this compound called adrenolutin has been isolated and characterized very well [48]. A similar compound may be formed from norepinephrine. Enolization of the ketocatechol and its further oxidation generates again polymeric melanin pigments (Figure 8). In mammalian substantia nigra, such melanin formation is possible. However, it is not clear if any such melanin is produced from epinephrine and norepinephrine in any insect tissues.

**Figure 9:**
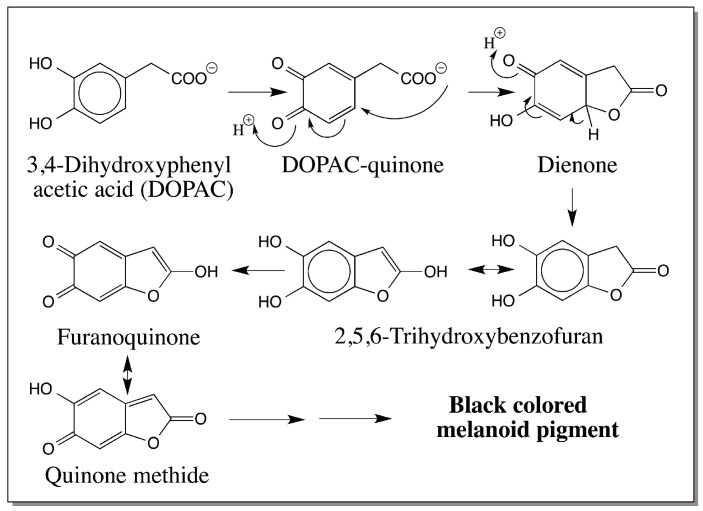
Oxidative cyclization of 3,4-dihydroxyphenylacetic acid. The quinone formed by the oxidation of 3,4-Dihydroxyphenylacetic acid among other reactions exhibits an intramolecular cyclization and produces a trihydroxybenzofuran, which undergoes oxidative polymerization yielding a black colored melanoid pigment.

Finally, there is one possible minor pathway for melanoid pigment production from a derivative of dopamine. 3,4-Dihydroxyphenylacetic acid is produced in a number of biological systems as the degradation product of dopamine. Being an *o*-diphenol, it is also easily oxidized practically by all tyrosinases and phenoloxidases. The quinone product exhibits three different reactions of which intramolecular cyclization parallels dopamine melanin formation [50]. Its rapid intramolecular cyclization generates 2,5,6-trihydroxybenzofuran. This reactive trihydric phenol is further oxidized to furanoquinone and gets converted to a transient quinone methide (Figure 9). Eventually all these reactive compounds polymerize and produce a black colored insoluble melanoid pigment [50]. 3,4-Dihydroxyphenylacetic acid is produced as a degradation product of dopamine in mammalian as well as insect systems. However, whether the melanoid product derived from 3,4-dihydroxyphenylacetic acid is formed in any biological system or not remains to be determined. Certainly more work is needed on this and other catecholamine derivatives destined for oxidative polymerization.

## 10. Pheomelanin

**Figure 10:**
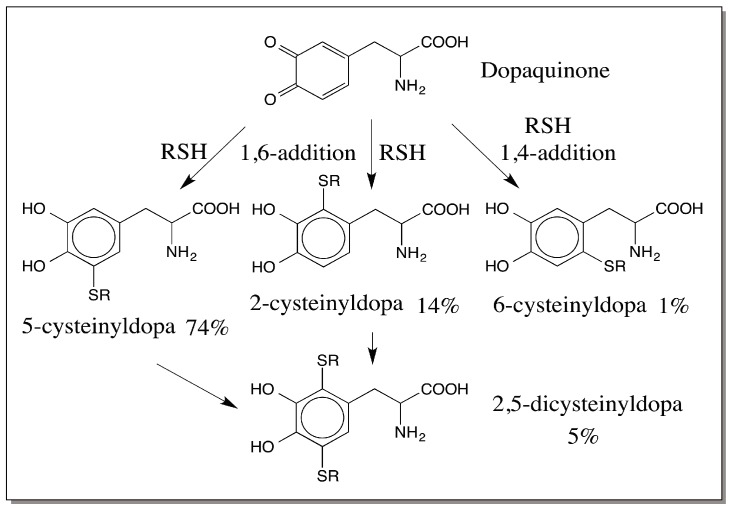
Thiol addition to dopaquinone. Thiols such as cysteine add on to dopaquinone not by conventional Michael-1,4-addition reaction. They react by an unconventional 1,6-addition, producing 5-*S*-cysteinyl dopa as the major product and 2-*S*-cysteinyldopa as the minor product. In addition 2,5-dicysteinyldopa is also formed to a certain extent. RSH—Cysteine.

Pheomelanin is a branch point in the overall melanin biosynthetic process [2,3,5,8,9,42]. Dopaquinone as well as dopamine quinone that are formed by the action of tyrosinase exhibit instantaneous nonenzymatic addition reactions with endogenous thiols such as cysteine producing thiolated catecholamines. These thiolated catecholamines are the building blocks of pheomelanin. The availability of thiols in any system will dictate how much pheomelanin will be synthesized in a given tissue. Tissues rich in reduced glutathione or cysteine naturally favor the production of thiolated catecholamine derivatives and hence pheomelanin. Tissues poor in thiols will not be able to synthesize significant amounts of pheomelanin. Thiols add onto quinones by a quite strange reaction and do not follow the typical nucleophilic addition rules. Thiols produce uniquely 1,6-addition products rather than 1,4-addition products, as shown in Figure 10 [42,51,52,53].

**Figure 11:**
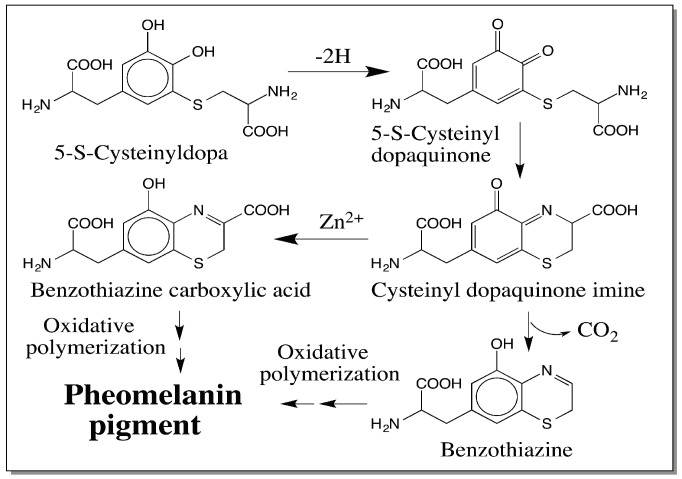
A simplified model for the biosynthesis of pheomelanin. Using the major product—5-*S*-cysteinyldopa—as the precursor, the biosynthesis of pheomelanin is illustrated in this figure. Oxidation of 5-*S*-cysteinyldopa produces its quinone, which undergoes internal condensation reaction producing the quinone imine. Quinone imine either rearranges to form benzothiazine-3-carboxylic acid or undergoes decarboxylation to benzothiazine. Oxidative polymerization of these two compounds produces the majority of the red to brown pheomelanin pigment in all animals.

The thiolated catechols are further oxidized to their corresponding quinones most likely by the endogenous dopaquinone. The cysteinyl amino group is situated in a suitable position to form a cyclic adduct. This reaction gives rise to a quinone imine that could either undergo decarboxylative aromatization or simple isomerization coupled aromatization producing the benzothiazine and its 3-carboxylic derivative respectively [2,3,5,8,9,42,51,52,53]. Oxidative polymerization of these two derivatives leads to the majority of the red to brown colored pheomelanin pigments observed in animals (Figure 11).

Mammalian systems produce significant amounts of pheomelanin [42,43]. In contrast, the reports on the presence of pheomelanin in invertebrate animals and especially insects are very few [54]. Insects with their poor reserves for thiols may produce much less pheomelanin. Insects make melanin for a variety of reasons and it is extremely important for their successful survival. However, studies aimed at differentiating between eumelanin and pheomelanin in insects are few and far between. First proposal that pheomelanin could be formed in insects was pointed out by Latocha et al. [55]. These authors performed pyrolytic gas chromatography—mass spectrometry analysis of melanin from black, gray and yellow colored *Drosophila melanogaster* and found the presence of pheomelanin related compounds in the yellow strain. More recently, Galván et al. [54] provided the first concrete evidence for the presence of pheomelanin in insects. They performed Raman spectroscopic studies on the grasshopper and revealed the presence of eumelanin in black regions of cuticle and pheomelanin in the yellowish red region of the cuticle. Moreover, HPLC analysis of the degradation products from cuticular melanin revealed the presence of cysteinyldopamine derived pheomelanin products thereby confirming the production of pheomelanin in insects. Pheomelanin has also been identified recently in parasitic wasps [56]. The vertebrate pheomelanin found in the peripheral tissues are usually made up of cysteinyldopa units [54], where as the neuromelanin may contain both dopa and dopamine derived pheomelanins [54]. Interestingly, insect pheomelanin seems to be derived mostly from cysteinyldopamine rather than cysteinyldopa. It would be interesting to pursue the role of pheomelanins in insects especially in the host-defense mechanisms and cuticular hardening reactions. Certainly, more studies are needed to shed light on the presence, physiological role and biosynthesis of pheomelanin in insects.

## 11. Insects Use Melanization and Sclerotization for Cuticular Coloration

**Figure 12:**
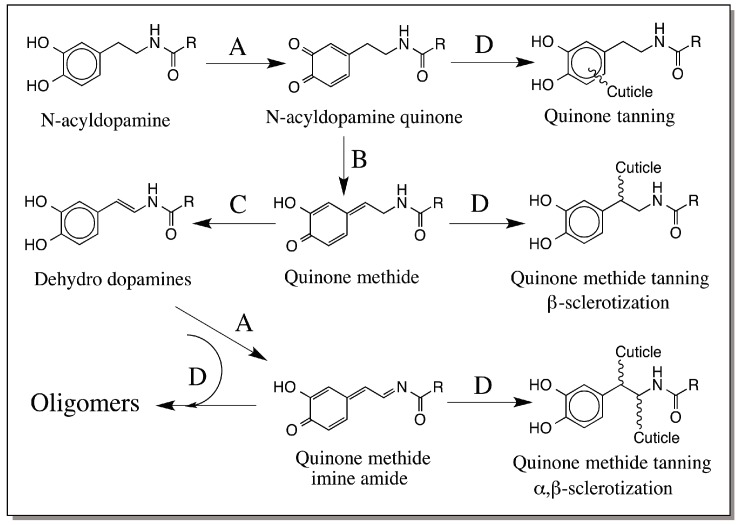
Unified mechanism for sclerotization of insect cuticle. Sclerotizing precursors such as *N*-acetyldopamine (R = CH_3_) and *N*-β-alanyldopamine (R = CH_2_CH_2_NH_2_) are oxidized by cuticular phenoloxidases (A) to their corresponding quinones, which participate in quinone tanning reaction by forming Michael-1,4-addition reaction with cuticular nucleophiles. Quinone isomerase (B) converts part of the quinones to quinone methides and provide for quinone methide tanning through Michael-1,6-addition reactions. Some of the quinone methide also serves as substrate for quinone methide isomerase (C), which transforms them to 1,2-dehydro-*N*-acyldopamines. Oxidation of dehydro compounds by phenoloxidase generates the bifunctional quinone methide imine amides that form adducts with both the side chain carbon atoms. Some of the dehydro compound also undergoes oligomerization reaction (D = nonenzymatic reactions).

In insects, a process that is very much similar to melanization occurs simultaneously and independently. This process called sclerotization, is extremely vital for the survival of most insects. It aids the hardening of their exoskeleton (=cuticle) and protects the soft bodies at various stages of insect’s life [57,58,59,60,61]. Unhardened cuticle allows dehydration and desiccation of the insect, and is susceptible to easy invasion by foreign organisms [57,58,59,60,61]. Hardened cuticle apart from protecting the insects against external harsh elements serves as a point for attachment of muscles and other organs. Therefore hardening of the cuticle is an indispensable process for practically all insects. Our laboratory has been investigating the molecular mechanism of sclerotization for over three decades and has clarified the molecular mechanisms of cuticular sclerotization, which is summarized in Figure 12 [4,57,58,59].

At the beginning of sclerotization, cuticular phenoloxidases oxidize catecholamine derivatives such as *N*-acetyldopamine (NADA) and *N*-β-alanyldopamine (NBAD) to their corresponding quinones. These quinones may act as gluing agents and bond to cuticular components most notably, chitin and structural proteins [57,58,59,60,61], or serve as substrates for the next enzyme in the metabolic pathway called quinone isomerase [62,63,64]. Quinone isomerase converts 4-alkylquinones to hydroxyquinone methides and provide them for: (a) quinone methide tanning where in the side chain of the catecholamine forms adducts with the regeneration of the catecholamine ring; or (b) the next enzyme in the pathway—quinone methide isomerase [63,65,66]. Quinone methide isomerase acts on unstable NADA and NBAD quinone methides and converts them to 1,2-dehydro-*N*-acyldopamine derivatives [63,65,66]. Further oxidation of dehydro dopamine derivatives produces the reactive quinone methide imine amides that crosslink proteins and chitin or react with parent catechols forming dimers, trimers and other oligomers [67,68,69].

In many insects, sclerotization is associated with coloration of cuticle, which is either due to melanin or due to quinonoid end products of sclerotizing catechols or a combination of both [59,60]. Cuticles using NBAD are often brown colored, which may have both melanin and colored sclerotin end products [59,60]. On the other hand, cuticles using NADA are often colorless but are hardened by sclerotization reactions [60]. Both sclerotization and melanization are two different biochemical processes with different physiological roles and are used for entirely different purposes. However, there exist many similarities in these two processes [57]. Both processes are initiated by the enzyme, phenoloxidase or tyrosinase, which oxidizes the catecholic precursors to their corresponding quinones. In sclerotization, the quinone cannot undergo intramolecular nucleophilic addition reaction as the amino group is protected by acylation. Therefore the reaction is diverted to external nucleophiles. However, in melanogenic process, the quinone exhibits intramolecular cyclization due to the presence of appropriately positioned amine group. The next set of reactions to be considered is the transformation of quinone to quinone methide. In sclerotization, quinone isomerase performs the conversion of *N*-acyldopamine quinones to quinone methides [62,63,64]. The parallel reaction in melanogenesis is catalyzed by DCT that converts dopachrome to quinone methide [57]. The next reaction in sclerotization is the conversion of quinone methide to 1,2-dehydro-*N*-acyldopamine catalyzed by the enzyme, quinone methide isomerase [63,65,66]. The similar reaction in melanogenesis may be assisted by DCT itself, or occur via nonenzymatic conversion as the driving force for such a reaction could come from the aromatization of quinonoid nucleus to benzenoid structure [57]. In both cases, the side chain of the catecholamine is specifically dehydrogenated to produce a desaturated compound. Generation of side chain desaturated compound is absolutely essential for both the processes as it provides additional loci for adduct and/or crosslink formation. Towards the end, the oxidation of desaturated catechols to its quinonoid products takes place. In sclerotization, phenoloxidase or even nonenzymatic reactions could aid the oxidation process. In melanization DHICA oxidation is aided by DHICA oxidase, while DHI oxidation may be tyrosinase catalyzed or nonenzymatic in nature [36,37,38]. These oxidations produce in both cases, oligomeric products before polymers are generated. In the case of dehydro NADA, we have demonstrated the production of not only dimers and trimers, but also oligomers up to hexamers by mass spectral studies [69]. Similarly, for both DHICA and DHI, oligomeric products have been characterized [70,71,72,73,74].

In spite of such remarkable similarities, significant differences exist between melanin biosynthesis and sclerotization reactions [4,57,58,59]. The reactivity of the catecholamines in melanin biosynthesis is mostly confined to internal cyclization and self-polymerization, although nucleophiles from proteins and other molecules are also incorporated at later stages. However the reactivity of catecholamine derivatives in sclerotization are mostly directed to external reactivities with other nucleophiles with the exception of some oligomerization reaction. In other words, catecholamines are used to glue and bond structural proteins and chitin polymers together to form a supramolecular structure in the case of cuticular sclerotization [57,58,59,60,61]. On the other hand, in melanin biosynthesis, the majority of the catecholamines are used for the production of polymeric melanin pigment [2,3,5,9,42]. Thus the quinone products formed in sclerotization are destined for external reactivities, while the quinones generated during melanogenesis are mostly used for internal reactivity with the exception of pheomelanogenesis. In this instance, dopaquinone and related compounds form adducts with external sulfur nucleophiles forming thiolated catechols. Finally, a note on the enzymes associated with the initiation of these two processes. The insect tyrosinases do not have any significant homology with the mammalian tyrosinases [75]. In insects, the sclerotization process is initiated by laccase, which is a *p*-diphenoloxidase rather than tyrosinase, which is a typical *o*-diphenoloxidase [76]. Laccases oxidize both *o*-diphenols and *p*-diphenols. Tyrosinases do not oxidize *p*-diphenols but do oxidize *o*-diphenols. Tyrosinase uses a two-electron oxidation process to generate the quinone directly from their catecholic substrates. Laccases on the other hand, use one-electron oxidation producing semiquinone as the primary products [77]. The semiquinone, however, undergoes disproportionation reaction to form the parent diphenol and quinone product. Thus, both tyrosinases and laccases, which are structurally unrelated to each other, generate the same quinonoid product from their diphenolic substrates.

There are major differences in melanin biosynthesis that occurs in mammalian system and insect systems [4,57]. Melanin biosynthesis in mammals is limited mostly to specialized cells called melanocytes [2]. All the enzymes associated with melanin biosynthesis are confined to this organelle. Often these enzymes are membrane bound, inextractable and form a tight complex with one another and with other melanogenic related proteins [17]. In insects, melanin is made both in the exoskeleton and inside the body. The cuticular phenoloxidases tend to be cuticle-bound enzymes while, hemolymph phenoloxidases are of soluble type. The enzymes associated with insect melanogenesis are present in their hemolymph (=blood), which bathes the entire insect body and hence these enzymes are distributed through out the insect body [4,57]. Tyrosinase is present in its active form in melanocytes. However, having the active phenoloxidase in the insect hemolymph is very deleterious to the insects, as its substrates are also present in the circulating blood and hence they are subjected to oxidation [4,57]. This interaction can result in the production of undesired quinonoid toxic compounds. Hence insects keep the phenoloxidase in the inactive proenzyme form and activate it during a specific need. As stated early, mammalian tyrosinase performs both the oxidation of tyrosine and dopa to dopaquinone. However, insect phenoloxidases do not seem to possess much of “tyrosine hydroxylase” activity. Insects mostly use specific tyrosine hydroxylase for dopa production. There are significant differences at the stage of dopachrome conversion reaction also [4,57]. Mammalian DCT converts dopachrome to DHICA [18,19,20]. However, insect DCDT that were characterized so far, only produce DHI as the sole product [4,21,22,23,46,57]. Whether nonenzymatically dopachrome is converted to DHICA or not in insects is yet to be assessed. As a consequence, insects may be specifically making DHI melanin [78] and not the DHICA or mixed melanin as is observed in mammalian melanogenic process. Eumelanin thus seems to be the dominant melanin present in the insects [54]. Even though the presence of pheomelanin in three insect systems has been reported, its role and abundance in insect is yet to be determined [54,55,56].

## 12. Importance of Melanin for Insect Biochemistry and Physiology

Insects use melanin biosynthesis for a variety of physiologically important processes that are extremely vital for their survival [4,57,58,59,60,61,79,80]. Cuticular melanization is one of the important processes. As stated earlier, both sclerotization and melanization often occur in the same cuticle to give different color and strength to the cuticle. Insects have developed an amazing array of coloring pattern for their body to help them conceal against specific backgrounds in different environments for better survival. The coloring patterns developed over a period of several generations by natural selection process have helped them to avoid predation. During industrial revolution, pepper moths evolved darker coloration of cuticle to avoid predation on trees coated with darker industrial pollutants. This process referred to as industrial melanism is a classical case for evolution of color variation [79,80].

Another physiological process where melanin plays an essential role is wound healing/sealing reactions [81,82,83,84]. During wounding, massive deposition of melanin occurs at the wound site in insects for two major reasons. One it helps to seal the wound along with other biochemical processes to prevent further blood loss. Second, melanogenic quinonoid products are well known cytotoxic compounds. Therefore, they will serve to kill any opportunistic microorganisms trying to enter the insect body through the wound site [81,82,83,84].

Melanin also plays a third important role in host defense reactions or innate immune responses [4,57,58,59,60,61,81,82,83,84,85,86]. Higher animals have evolved an elaborate and complex immune system that can exhibit both memory and specificity to recognize and respond to various infectious agents. For example, they use lymphocytes and specific antibodies to ward of infections successfully. Lacking such vital immune response components, insects have managed to defend themselves against invading pathogens, which gain access into the insect by a plethora of host defense reactions. Melanization is an important and indispensable component of this innate immune responses [4,57,58,59,60,61,81,82,83,84,85,86]. Insects use a wide range of cellular and humoral defense reactions to protect themselves. The cellular responses are phagocytosis, nodule formation and encapsulation, while the humeral responses are antibacterial peptide synthesis production, use of lectin type molecules and activation of prophenoloxidase cascade. Organisms entering the insects are often found encapsulated and melanized. Therefore, phenoloxidase and melanin are considered as important components of insect’s immune system. This process limits the damage caused by the intruder. Again, the cytotoxic quinonoid products formed during the process also help the insect to fight the invading organisms. A lot of review articles have appeared in the literature time to time on this topic [4,81,82,83,84,85,86]. The rest of the article will focus mainly on the cuticular pigmentation and melanization, as it has become an intensive area of research in recent years.

## 13. Insect Genes Associated with Melanization and Cuticular Sclerotization

**Figure 13:**
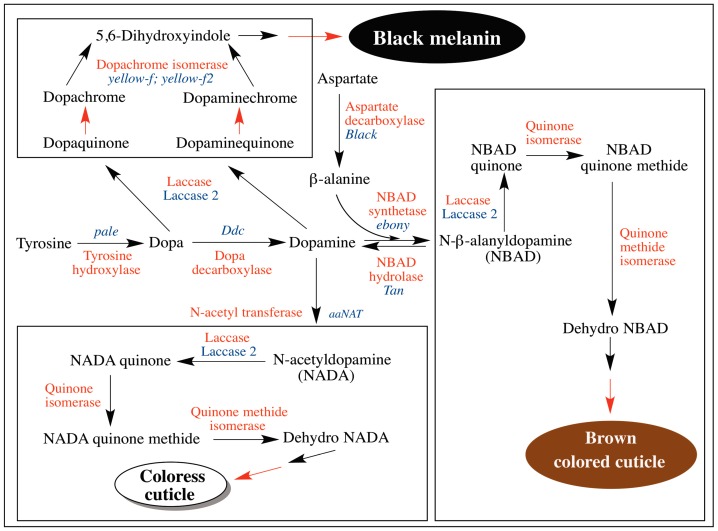
Genes associated with melanization and sclerotization processes in insect cuticle. Insect cuticular melanogenesis is intimately linked to cuticular sclerotization process, which makes the cuticle strong and hard by crosslinking cuticular proteins and chitin. Both processes start with the amino acid, tyrosine. Tyrosine hydroxylase converts tyrosine to dopa. Dopa decarboxylase generates dopamine from dopa. Both dopa and dopamine can serve as the precursor for melanin. Cuticular laccase seems to be responsible for melanization in cuticle by oxidizing these two catechols to quinones. Both these quinones after conversion to chromes serve as substrates for DCDT, which produce DHI as the product. Oxidative polymerization DHI makes the black colored melanin pigment in cuticle. Most importantly, dopamine is primarily converted to NBAD and NADA by the action of NBAD synthetase and *N*-acetyl transferase respectively. Oxidation of these catechols produces their quinones, which are acted on by quinone isomerase and quinone methide isomerase sequentially leading to the generation of dehydro compounds. Oxidative coupling of dehydro compounds along with the adduct formation by *N*-acyldopamine quinones and quinone methides account for sclerotization reactions. (Enzymes catalyzing various reactions are labeled in red. The genes associated with these enzymes are named in blue. Black arrow enzyme catalyzed reactions; red arrow nonenzymatic reactions).

Several genes are associated with cuticular hardening and sclerotization pathways. However, only some of the important genes that are directly involved in these two processes are shown in the Figure 13 and only these will be discussed. Thus far, eight genes have been identified to play a crucial role in insect cuticular pigmentation and sclerotization process. These are *pale*, *DDC*, *aaNAT*, *ebony*, *tan*, *black*, *Laccase 2*, and *yellow* (*f* and *f2*), which code for tyrosine hydroxylase, dopa decarboxylase, arylalkylamine-*N*-acetyltransferase, NBAD synthetase, NBAD hydrolase, aspartate decarboxylase, laccase associated with cuticular sclerotization, and DCDT, respectively. The genes for quinone isomerase and quinone methide isomerase associated with cuticular sclerotization are yet to be identified.

The hydroxylation of tyrosine to dopa is the first step involved in the biosynthesis of the catecholamines. This reaction is specifically catalyzed by tyrosine hydroxylase (TH) encoded by the *pale* gene [87]. Tyrosine hydroxylase is an iron containing monooxygenase incorporating one atom of molecular oxygen into tyrosine and reducing the other atom to water with the help of tetrahydrobiopterin cofactor [88]. The enzyme contains an amino terminal hydrophilic regulatory domain that has several phosphorylation sites and a carboxy terminal hydrophobic domain, which has catalytic site and cofactor binding site [88]. In *Drosophila* alternative splicing of the primary transcript produces two isoforms *TH1* and *TH2*, which are expressed in different tissues [87]. They also have different phosphorylation sites, and different kinetic parameters. TH1 is expressed in the central nervous system and is activated by phosphorylation. This isoform is mainly associated with the production of catecholamine derivatives distained for neurotransmission. Isozyme TH2 is expressed in epidermis and is responsible for the production of dopa needed for cuticular sclerotization and melanization [87]. Importantly, tyrosine hydroxylase is required for the survival of *Drosophila* as embryos that are null mutant of *pale* are unpigmented and fail to hatch. Similarly, its essential role for the survival of the silkworm, *Bombyx mori* has also been documented [89].

The *DDC* gene codes the next important enzyme, dopa decarboxylase. It is a pyridoxal-5′-phosphate containing enzyme, playing a pivotal role in the biosynthesis of neurotransmitters, dopamine, and serotonin by specifically decarboxylating dopa and 5-hydroxytryptophan, respectively. Dopamine is absolutely essential for the biosynthesis of both melanin and sclerotin in insects [57,58,59,60,61,78,79,80,81]. *Drosophila* has five aromatic-decarboxylase genes of which, only one is responsible for the catalyzing the decarboxylation of dopa. Two *DDC*-like genes are characterized as α-methyl-dopa resistant proteins [90] and the two other are identified as tyrosine decarboxylase genes [91]. The catalytically active form of dopa decarboxylase exists as a homodimer and is expressed in the epidermis. At the active site, the enzyme has a H192, which is associated with pyridoxal phosphate binding and a Thr82 that is involved in substrate binding [92]. Loss of function at the *DDC* locus by mutation is often lethal. The cuticle of surviving flies is very soft and mostly unpigmented, a phenotype that emphasizes the vital need of dopamine for both sclerotization and melanization reactions.

The *aaNAT* gene encodes arylalkylamine-*N*-acetyltransferases. In mammals, this protein catalyzes the essential first step in the formation of melatonin in the pineal gland and is associated with circadian rhythm. In insects, the aaNAT enzymes have different physiological roles [93,94]. They catalyze the formation of one of the two widely used insect cuticular sclerotizing precursors, NADA. They are also important for the inactivation of a number of biogenically produced monoamines (norepinephrine, dopamine, octopamine) due to the fact that insects do not possess monoamine oxidase for such inactivation. Acetyl CoA is the co-substrate for aaNAT in producing the *N*-acetyl derivatives of amines [95]. Recent studies point out that aaNAT suppresses melanin pigmentation in some insect cuticle [93,94,95,96,97,98,99]. The adult melanism (*mln*) mutant of silkworm *Bombyx mori* exhibits a strong black coloration in contrast to the wild type’s normal coloration [96]. Studies indicate that this black coloration correlates with loss of aaNAT function in *mln* gene. In the *mln* larvae, only the regions that are normally heavily sclerotized show an abnormal pigmentation. All these results point that aaNAT has an important role in normal coloration of cuticle and its loss of function by mutation causes possible build up of excess dopamine that is then diverted for dopamine melanin production [96,97].

NBAD synthetase coded by the *ebony* gene is expressed in the epidermal cells and plays a major role in the tanning process by providing the crucial sclerotizing precursor, NBAD [100]. The ebony gene coding for this enzyme is also responsible for the biosynthesis of carcinine (β-alanylhistamine) by the condensation of β-alanine and histamine in the nervous system mainly in the optic ganglia cells [100]. NBAD synthetase appears to be related to the family of the microbial non-ribosomal peptide synthetases and has three functional domains: activation domain, thiolation domain, and putative amine-selecting domain [100]. The reaction catalyzed by NBAD synthetase requires ATP, β-alanine and dopamine. The coupling reaction resembles very much the non-ribosomal peptide synthesis [100]. First the inactive form of the apoenzyme has to be converted to active holoenzyme form by the addition of 4-phosphopantetheinyl group of coenzyme A on to the conserved S611 present at the thiolation domain of the enzyme by 4′-phosphopantetheinyl transferase. The thiol group thus introduced into the holoenzyme plays a pivotal role in the catalysis. At the activation domain, β-alanine is adenylated by ATP and the resultant activated β-alanine is transferred to the thiol group. The terminal group of dopamine bound at the amine-selecting domain then attacks the carbonyl group of β-alanine present at the thiolation site resulting in the biosynthesis of NBAD [100]. *Ebony* mutant flies have a very soft cuticle with a very dark color [101]. Since NBAD is not synthesized in the mutant flies, their cuticular sclerotization is dramatically affected. The unused dopamine is shunted for melanin production and hence these flies have a dark colored but soft cuticle.

Tan is another crucial gene associated with the melanogenic pathway in the cuticle [102,103]. This gene codes for NBAD hydrolase, which is responsible for releasing dopamine back from NBAD. This ensures the sufficient utilization of NBAD for cuticular sclerotization and then allowing the remaining NBAD be converted back to dopamine for melanin biosynthesis. Therefore most likely the melanin made in cuticle is dopamine melanin and not dopa melanin. This is in sharp contrast with the melanin made in the skin of higher animals, which is mainly dopa-derived melanin. In the photoreceptor neurons, NBAD hydrolase has a different role by hydrolyzing carcinine and regenerating histamine necessary for normal vision [102]. NBAD hydrolase closely resembles a family of cysteine peptidases found in fungi. Notably it exhibits high sequence similarity to isopenicillin-*N*-acyltransferase, which is responsible for the synthesis of penicillin G. This enzyme possesses two activities—a hydrolase and an acylase activity [102]. NBAD hydrolase activity is similar to the first one. In addition, NBAD hydrolase processing is also similar to isopenicillin-*N*-acyltransferase processing. Thus, the *tan* gene product is made as an inactive protein, which is self-processed at Gly121-Cys122 to form α,β subunits. Neither of them individually are active but the heterodimeric α,β-pair is biologically active [103].

The black gene encodes for aspartate decarboxylase, which catalyzes the formation of β-alanine from aspartate [104]. β-alanine is required for the formation of the sclerotizing precursor, NBAD by the ebony gene. It should be noted here that β-alanine can also be formed by uracil hydrolysis and that some insects depend on this pathway in supplying β-alanine at the larval stage. However, the majority of β-alanine seems to arise by the action of aspartate decarboxylase [105]. Association of β-alanine to insect pigmentation was known for a long time, but only recently, the molecular biological aspects associated with the process are coming to light. The knockdown of aspartate decarboxylase by double stranded RNA in the beetles *Tribolium castenium* led to the formation of dark cuticle compared to the rust-red color wild type [105]. Similarly, black pupal mutant in the silkworm, *Bombyx mori* was shown to be due to frame shift mutation in aspartate decarboxylase gene [106]. Therefore, similar to NBAD synthetase mutant, black mutant flies exhibit the dark black color phenotype due to the accumulation of dopamine that is not used for NBAD synthesis but is used subsequently for dopamine melanin synthesis.

Laccase belongs to the group of multicopper oxidases found in a variety of organisms [107,108]. They carry out one electron oxidation of their substrates and use the extracted electrons to reduce molecular oxygen into water. Laccases have four copper ions, which are distributed in two different sites. The type 1 site (T1) carries one copper ion and is responsible for substrate oxidation. The type 2 site (T2/T3) has tricopper cluster and is associated with oxygen reduction [107,109]. Both *o*-diphenoloxidases and laccases are responsible for melanogenesis in insects. The former is probably responsible for melanization observed in the blood and wounding sites while the latter is involved in pigmentation and sclerotization of cuticle. Recent studies show that laccases are vital for the oxidation of catecholic compounds in insect cuticle [76,108,110,111,112,113]. The RNAi experiments carried out in *Tribolium castenium* attested the key role played by laccase 2 in sclerotization reaction in this beetle [76]. The presence of two spliced isoforms of laccase (laccase 2A and laccase 2B) has been reported in many insects including *Manduca sexta* [110], *Tribolium castaneum* [112], *Anopheles gambiae* [112], and *Bombyx mori* [113]. Interestingly, the laccase 2 isoforms exhibit no significant differences in their substrate specificity [112]. Futahashi et al. [111] demonstrated that laccase 2 and not tyrosinase 1 or 2 catalyzes the first step in both sclerotization and melanization pathways operating in the cuticle of stinkbugs. Insects injected with dsRNA for tyrosinase 1, tyrosinase 2, and laccase1 had pigmented and sclerotized cuticle and had no lethality whereas the ones injected with dsRNA for Laccase 2 had a soft and colorless cuticle and their adults died within few days after eclosion [111]. The purification and characterization of cuticular laccases has presented considerable challenges to scientists working in the area. Often cuticular laccases are tightly bound and require proteolytic treatment to release [113]. Yatsu and Asano [113] reported that cuticular laccase from *Bombyx mori* is extractable with urea but requires activation by trypsin. Thus, it is possible that the cuticular laccases are present in the inactive zymogen form and need activation by proteases. However, studies by Dittmer et al. [110] on the recombinant *Manduca sexta* protein show that the laccase is active with out protease treatment.

DCDT is one of the important enzymes of melanogenesis. DCDT has been purified and characterized from many insects since it was first discovered in *Manduca sexta* [4,21,22,23,46,57,58,59]. Importantly, DCDT is also associated with the innate immune response of insects like tyrosinase [4,114,115,116,117]. Activity of DCDT increases in response to microbial infections in *Drosophila melanogaster* [116] and injection of Sephadex beads into mosquitoes [114]. The infection of *A. gambiae* by malarial parasite causes an increase in the transcripts of DCDT [117]. DCDT belongs to a group of proteins called yellow gene family [118]. In *Drosophila melanogaster*, fourteen yellow related genes have been recognized. The first yellow gene identified is associated with cuticular melanization and is named *yellow-y* [118]. The *yellow-y* gene has been extensively studied but the function of the protein encoded by this gene remains still undetermined. Yellow protein is a secreted protein that has emerged from Royal Jelly protein and seems to be multifunctional [118]. It is expressed in larval salivary gland, in the nervous system of adults, and is associated with the black pigmentation in the adult flies. Mutation in this gene is responsible for the formation of yellow to brown coloration of the cuticle and hence the name yellow [118]. There are many hypotheses regarding the function of the yellow-y protein and how it promotes dark pigmentation in the literature. However many of these suggestions are not supported by experimental verifications. Walter et al. [119] evoked that it is a structural protein that is required for crosslinking DHI. However, to date even in mammalian system, no one has identified such a functional protein. Wittkopp et al. [80,120,121] suggested that yellow protein works downstream of dopa in melanin biosynthesis but the site of action still remains a mystery. Han et al. [46] expressed the yellow-y protein successfully in insect cell line, but were unable to provide support that it catalyzes DCDT reaction. However, their studies successfully identified *yellow-f* and *yellow-f2* as DCDT [46]. Drapeau [122] proposed that *yellow-y* is a hormone or growth factor like protein that induces melanin formation by activating a signaling pathway. However, again experimental evidence for such a proposal is still missing. Thus, in spite of the enormous work carried out on the yellow gene family, its relation to cuticular melanogenesis remains still unexplained. Further studies are desperately needed to resolve this aspect.

## 14. Summary and Conclusions

A simplistic view of melanogenesis was delineated in the early part of last century by the pioneering work of Raper and Mason. Extensive studies carried out by numerous laboratories resulted in the discovery of several proteins and enzymes associated with the melanogenic pathway. Many research groups elucidated the function of many genes encoding the regulatory proteins and/or enzymes involved in the pigmentation pathway. Intensive investigations on the biochemistry and mechanism of transformation of various catecholamine derivatives resulted in the identification of novel reactive transient intermediates. From these studies, a clear and detailed picture has emerged not only on the biochemical aspects but also on the molecular biological aspect of melanogenesis. We are beginning to see some unity and variations in the scheme of reactions used by this process in different groups of organisms. Some important uncertainties are still unresolved. How is DHI biosynthesized in higher animals? Do insects and other arthropods make significant amounts of pheomelanin and DHICA melanin? Why are two different types of dopachrome converting enzymes present in the two major groups of animals? How are various enzymes regulated to produce different shades of color? Are there any new enzymes/proteins to be discovered? Since melanin production in animals is associated with a number of pathological conditions, more research is certainly needed. Unraveling the role of many genes associated both with both the melanogenic process and sclerotinogenic process could be extremely helpful to treat some of the associated diseases.

Finally, a note on the hormonal regulation of melanogenesis in mammalian and insect systems warrants some attention. We are beginning to understand some aspects of hormonal regulation of melanogenesis in mammalian system [123,124,125]. Even melanogenic precursors, tyrosine and dopa seem to act as hormone-like regulators of melanogenic pathway in mammals [126]. However, in insects, the hormonal regulation of both melanogenic and sclerotinogenic processes need to be explored in details to shed more light on the physiological, biochemical and genetic aspects of these two processes. With the advent of molecular biology tools, we are certain that some of these aspects will be brought to light in the near future.

## Abbreviations

NADA	*N*-acetyldopamine
Dehydro NADA	1,2-Dehydro-*N*-acetyldopamine
DHI	5,6-Dihydroxyindole
DHICA	5,6-Dihydroxyindole-2-carboxylic acid
NBAD	*N*-β-alanyldopamine
Dehydro NBAD	1,2-Dehydro-*N*-β-alanyldopamine
DCT	Dopachrome tautomerase
DCDT	Dopachrome decarboxylase/tautomerase

## References

[1] Nicolaus R.A. (1968). Melanins.

[2] Prota G. (1992). Melanins and Melanogenesis.

[3] Ito S. (2003). A chemist’s view of melanogenesis. Pigment Cell Res..

[4] Sugumaran M. (2002). Comparative biochemistry of eumelanogenesis and the protective roles of phenoloxidase and melanin in insects. Pigment Cell Res..

[5] D’Ischia M., Wakamatsu K., Cicoira F., di Mauro E., Garcia-Borron J.C., Commo S., Galván I., Ghanem G., Kenzo K., Meredith P. (2015). Melanin and melanogenesis: From pigment cells to human health and technological applications. Pigment Cell Melanoma Res..

[6] Hearing V.J., Tsukamoto K. (1991). Enzymatic control of pigmentation in mammals. FASEB J..

[7] Plonka P.M., Grabacka M. (2006). Melanin synthesis in microorganisms—Biotechnological and medical aspects. Acta Biochim. Pol..

[8] Galván I., Solano F. (2016). Bird integumentary melanins: Biosynthesis, forms, function and evolution. Int. J. Mol. Sci..

[9] Land E.J., Ramsden C.A., Riley P.A. (2004). Quinone chemistry and melanogenesis. Methods Enzymol..

[10] Raper H.S. (1926). The tyrosinase-tyrosine reaction: Production of l-3,4-dihydroxyphenylalanine from tyrosine. Biochem. J..

[11] Raper H.S. (1928). The aerobic oxidases. Physiol. Rev..

[12] Raper H.S. (1938). Some problems of tyrosine metabolism. J. Chem. Soc..

[13] Mason H.S. (1948). The chemistry of melanin. III. Mechanism of the oxidation of dihydroxyphenyalanine by tyrosinase. J. Biol. Chem..

[14] Mason H.S. (1955). Comparative biochemistry of the phenolase complex. Adv. Enzymol..

[15] Korner A.M., Pawelek J. (1980). Dopachrome conversion factor: A possible control point in melanin biosynthesis. J. Investig. Dermatol..

[16] Pawelek J., Korner A., Bergstrom A., Bologna J. (1980). New regulators of melanin biosynthesis and the autodestruction of melanoma cells. Nature.

[17] Orlow S.J., Zhou B.K., Chakraborty A.K., Druker M., Pifko-Hirst S., Pawelek J.M. (1994). High-molecular weight forms of tyrosinase and tyrosinase related proteins: Evidence for a melanogenic enzyme complex. J. Investig. Dermatol..

[18] Pawelek J. (1990). Dopachrome conversion factor functions as an isomerase. Biochem. Biophys. Res. Commun..

[19] Aroca P., Solano F., Garcia-Borron J., Lozano J. (1990). A new spectrophotometric assay for dopachrome tautomerase. J. Biochem. Biophys. Methods.

[20] Aroca P., Garcia-Borron J., Solano F., Lozano J. (1990). Regulation of mammalian melanogenesis I: Partial purification and characterization of a dopachrome converting factor: Dopachrome tautomerase. Biochim. Biophys. Acta.

[21] Aso Y., Kramer K.J., Hopkins T.L., Whetzel S.Z. (1984). Properties of tyrosinase and dopa quinone imine conversion factor from pharate pupal cuticle of *Manduca sexta*. Insect Biochem..

[22] Aso Y., Imamura Y., Yamasaki N. (1989). Further studies on dopa quinone imine conversion factor from the cuticles of *Manduca sexta* (L.). Insect Biochem..

[23] Sugumaran M., Semensi V. (1991). Quinone methides as new intermediates of melanin biosynthesis. J. Biol. Chem..

[24] Palumbo A., d’Ischia M., Misuraca G., de Martino L., Prota G. (1994). A new dopachrome-rearranging enzyme from the ejected ink of the cuttlefish, *Sepia officinalis*. Biochem. J..

[25] Palumbo A., d’Ischia M., Misuraca G., Prota G. (1987). Effect of metal ions on the rearrangement of dopachrome. Biochim. Biophys. Acta.

[26] Sugumaran M., Dali H., Semensi V. (1990). Formation of a stable quinone methide during tyrosinase catalyzed oxidation of α-methyldopa methyl ester and its implication in melanin biosynthesis. Bioorg. Chem..

[27] Costantini C., Crescenzi O., Prota G. (1991). Mechanism of the rearrangement of dopachrome to 5,6-dihydroxyindole. Tetrahedron Lett..

[28] Crescenzi O., Costantini C., Prota G. (1990). Evidence for the intermediacy of quinone-methides in the rearrangement of aminochromes to 5,6-dihydroxyindoles. Tetrahedron Lett..

[29] Kishida R., Saputro A.G., Kasai H. (2015). Mechanism of dopachrome tautomerization into 5,6-dihydroxyindole-2-carboxylic acid catalyzed by Cu(II) based on quantum chemical calculations. Biochim. Biophys. Acta.

[30] Wakamatsu K., Ito S. (1988). Preparation of eumelanin related metabolites, 5,6-dihydroxyindole, 5,6-dihydroxyindole-2-carboxylic acid, and their *O*-methyl derivatives. Anal. Biochem..

[31] Solano F., Martinez-Liarte J.H., Jimenez-Cervantes C., Garcia-Borron J.C., Lozano J.A. (1994). Dopachrome tautomerase is zinc containing enzyme. Biochem. Biophys. Res. Commun..

[32] Furumura M., Solano F., Matsunaga N., Sakai C., Spritz R.A., Hearing V.J. (1998). Metal ion binding specificities of the tyrosinase related proteins. Biochem. Biophys. Res. Commun..

[33] Jackson I.J., Chambers D.M., Tsukamoto K., Copeland N.G., Gilbert D.J., Jenkins N.A., Hearing V. (1992). A second tyrosinase related protein, TRP-2, maps and is mutated at the mouse *slaty* locus. EMBO J..

[34] Kroumpouzos G., Urabe K., Kobayashi T., Sakai C., Hearing V.J. (1994). Functional analysis of the *slaty* gene product (TRP2) as dopachrome tautomerase and the effect of a point mutation on its catalytic function. Biochem. Biophys. Res. Commun..

[35] Tsukamoto K., Jackson I.J., Urabe K., Montague P.M., Hearing V. (1992). A second tyrosinase related protein, TRP-2, is a melanogenic enzyme termed DOPAchrome tautomerase. EMBO J..

[36] Jimenez-Cervantes C., Solano F., Kobayasho T., Urabe K., Hearing V.J., Lozano J.A., Garcia-Borron J.C. (1994). A new enzymatic function in the melanogenic pathway: The 5,6-dihydroxyindole-2-carboxylic acid oxidase activity of tyrosinase-related protein-1 (TRP1). J. Biol. Chem..

[37] Kobayasho T., Urabe K., Winder A., Jimenez-Cervantes C., Imokawa G., Brewington T., Solano F., Garcia-Borron J.C., Hearing V.J. (1994). Tyrosinase-related protein-1 (TRP1) functions as DHICA oxidase in melanin biosynthesis. EMBO J..

[38] Olivares C., Jimenez-Cervantes C., Lozano J.A., Solano F., Garcia-Borron J.C. (2001). The 5,6-dihydroxyindole-2-carboxylic acid (DHICA) oxidase activity of human tyrosinase. Biochem. J..

[39] Lee Z.H., Hou L., Moellmann G., Kuklinska E., Antol K., Fraser M., Halaban R., Kwon B.S. (1996). Characterization and subcellular localization of human Pmel 17/silver, a 100-kDa (Pre)melanosomal membrane protein associated with 5,6-dihydroxyindole-2-carboxylic acid (DHICA) converting activity. J. Investig. Dermatol..

[40] Jimenez-Cervantes C., Martinez-Esparaza M., Solano F., Lozano J.A., Garcia-Borron J.C. (1998). Molecular interactions within the melanogenic complex: Formation of heterodimers of tyrosinase and TRP1 from B16 mouse melanoma. Biochem. Biophys. Res. Commun..

[41] Cooksey C.J., Garratt P.J., Land E.J., Pavel S., Ramsden C.A., Riley P.A., Smit N.P.M. (1997). Evidence of the indirect formation of the catecholic intermediate substrate responsible for the autoactivation kinetics of tyrosinase. J. Biol. Chem..

[42] Ito S., Wakamatsu K. (2008). Chemistry of mixed melanogenesis—Pivotal roles of dopaquinone. Photochem. Photobiol..

[43] Micillo R., Panzella L., Koike K., Monfrecola G., Nepolitano A., d’Ischia M. (2016). “Fifty shades” of black and red or how carboxyl group fine tune eumelanin and pheomelanin properties. Int. J. Mol. Sci..

[44] Zhang F., Dryhurst G. (1993). Oxidation of dopamine: Possible insights into the age-dependent loss of dopaminergic nigrostriatal neurons. Bioorg. Chem..

[45] Meiser J., Weindl D., Hiller K. (2013). Complexity of dopamine metabolism. Cell Commun. Signal..

[46] Han Q., Fang J., Ding H., Johnson J.K., Christenson B.M., Li J. (2002). Identification of *Drosophila melanogaster* yellow-f and yellow-f2 proteins as dopachrome-conversion enzymes. Biochem. J..

[47] Zuca F.A., Basso E., Cupaiolo F.A., Ferrari E., Sulzer D., Casella L., Zecca L. (2014). Neuromelanin of human substantia nigra: An update. Neurotox. Res..

[48] Palumbo A., d’Ischia M., Misuraca G., Prota G. (1989). A new look at the rearrangement of adrenochrome under biomimetic conditions. Biochim. Biophys. Acta.

[49] Manini P., Panzella L., Napolitano A., d’Ischia M. (2007). Oxidation chemistry of norepinephrine: Partitioning of the *o*-quinone between competing cyclization and chain breakdown pathways and their roles in melanin formation. Chem. Res. Toxicol..

[50] Sugumaran M., Duggaraju R., Jayachandran E., Kirk K. (1999). Formation of a new quinone methide intermediate during the oxidative transformation of 3,4-dihydroxyphenylacetic acids: Implications for eumelanin biosynthesis. Arch. Biochem. Biophys..

[51] Ito S., Prota G. (1977). A facile one step synthesis of cysteinyldopas using mushroom tyrosinase. Experientia.

[52] Napolitano A., Memoli S., Prota G. (1999). A new insight in the biosynthesis of pheomelanins: Characterization of a labile 1,4-benzothiazine intermediate. J. Org. Chem..

[53] Zhang F., Dryhurst G. (1994). Effects of l-cysteine on the oxidation chemistry of dopamine: Pathways of potential relevance to idiopathic Parkinson’s disease. J. Med. Chem..

[54] Galván I., Jorge A., Edelaar P., Wakamatsu K. (2015). Insects synthesize pheomelanin. Pigment Cell Melanoma Res..

[55] Latocha M., Chodurek E., Kurkiewicz S., Swiatkowska L., Wilczok T. (2000). Pyrolytic GC-MS analysis of melanin from black, gray and yellow strains of *Drosophila melanogaster*. J. Anal. Appl. Pyrolysis.

[56] Jorge Garcia A., Polidori C., Nieves-Aldrey J.L. (2016). Pheomelanin in the secondary sexual characters of male parasitoid wasps (Hymenoptera: Pteromalidae). Arthropod Struct. Dev..

[57] Sugumaran M. (1991). Molecular mechanisms for mammalian melanogenesis—Comparison with insect cuticular sclerotization. FEBS Lett..

[58] Sugumaran M. (1998). Unified mechanism for sclerotization of insect cuticle. Adv. Insect Physiol..

[59] Sugumaran M. (2010). Chemistry of cuticular sclerotization. Adv. Insect Physiol..

[60] Andersen S.O. (2010). Insect cuticular sclerotization: A review. Insect Biochem. Mol. Biol..

[61] Hopkins T.L., Kramer K.J. (1992). Insect cuticle sclerotization. Annu. Rev. Entomol..

[62] Saul S.J., Sugumaran M. (1989). *o*-Quinone: Quinone methide isomerase—A novel enzyme which prevents the destruction of self matter by phenoloxidase generated quinones during immune response in insects. FEBS Lett..

[63] Saul S.J., Sugumaran M. (1989). Characterization of a new enzyme system that desaturates the side chain of *N*-acetyldopamine. FEBS Lett..

[64] Saul S.J., Sugumaran M. (1990). 4-Alkyl-*o*-quinone/2-hydroxy-*p*-quinone methide isomerase from the larvae hemolymph of *Sarcophaga bullata*. I. Purification and characterization of enzyme catalyzed reaction. J. Biol. Chem..

[65] Saul S.J., Sugumaran M. (1989). *N*-Acetyldopamine quinone methide/1,2-dehydro-*N*-acetyldopamine tautomerase—A new enzyme involved in sclerotization of insect cuticle. FEBS Lett..

[66] Ricketts D., Sugumaran M. (1994). 1,2-dehydro-*N*-β-alanyldopamine as a new intermediate in insect cuticular sclerotization. J. Biol. Chem..

[67] Sugumaran M., Semensi V., Kalyanaraman B., Bruce J.M., Land E.J. (1992). Evidence for the formation of a quinone methide during the oxidation of the insect cuticular sclerotizing precursor, 1,2-dehydro-*N*-acetyldopamine. J. Biol. Chem..

[68] Sugumaran M. (2000). Oxidation chemistry of 1,2-dehydro-*N*-acetyldopamines: Direct evidence for the formation of 1,2-dehydro-*N*-acetyldopamine quinone. Arch. Biochem. Biophys..

[69] Abele A., Zheng D., Evans J., Sugumaran M. (2010). Reexamination of the mechanisms of oxidative transformation of the insect cuticular sclerotizing precursor, 1,2-dehydro-*N*-acetyldopamine. Insect Biochem. Mol. Biol..

[70] Kroesche C., Peter M.G. (1996). Detection of melanochromes by MALDI-TOF mass spectrometry. Tetrahedron.

[71] Pezzella A., Napolitano A., d’Ischia M., Prota G. (1996). Oxidative polymerization of 5,6-dihydroxyindole-2-carboxylic acid to melanin: A new insight. Tetrahedron.

[72] Napolitano A., Crescenzi O., Prota G. (1993). Copolymerization of 5,6-dihydroxyindole and 5,6-dihydroxyindole-2-carboxylic acid in melanogenesis: Isolation and of a cross-coupled product. Tetrahedron Lett..

[73] Ito S., Nicol J.A.C. (1974). Isolation of oligomers of 5,6-dihydroxyindole-2-carboxylic acid from the eye of the catfish. Biochem. J..

[74] Pezzella A., Panzella L., Natangelo A., Arzillo M., Napolitano A., d’Ischia M. (2007). 5,6-dihydroxyindole tetramets with “anomalous” interunit bonding patterns by oxidative coupling of 5,5′,6,6′-tetrahydroxy-2,7′-biindolyl: Emerging complexities on the way toward an improved model of eumelanin buildup. J. Org. Chem..

[75] Hall M., Scott T., Sugumaran M., Soderhall K., Law J.H. (1995). Proenzyme of *Manduca sexta* phenoloxidase: Purification, activation, substrate specificity of the active enzyme and molecular cloning. Proc. Natl. Acad. Sci. USA.

[76] Arakane Y., Muthukrishnan S., Beeman R.W., Kanost M.R., Kramer K.J. (2005). Laccase 2 is the phenoloxidase gene required for beetle cuticle tanning. Proc. Natl. Acad. Sci. USA.

[77] Nakamura T. (1960). On the process of enzymatic oxidation of hydroquinone. Biochem. Biophys. Res. Commun..

[78] Sugumaran M., Duggaraju R., Generozova F., Ito S. (1999). Insect melanogenesis II. Inability of *Manduca* phenoloxidase to act on 5,6-dihydroxyindole-2-carboxylic acid. Pigment Cell Res..

[79] True J.R. (2003). Insect melanism: The molecules matter. Trends Ecol. Evol..

[80] Wittkopp P.J., Beladade P. (2009). Development and evolution of insect pigmentation: Genetic mechanisms and the potential consequences of pleiotropy. Semin. Cell Dev. Biol..

[81] Sugumaran M., Soderhall K., Iwanaga S., Vastha G. (1996). Role of insect cuticle in immunity. New Directions in Invertebrate Immunology.

[82] Theopold U., Sschmidt O., Soderhall K., Dushay M.S. (2004). Coagulation in arthropods: Defense, wound closure and healing. Trends Immunol..

[83] Eleftherianos I., Revenis C. (2011). Role and importance of phenoloxidase in insect hemostasis. J. Innate Immun..

[84] Krautz R., Arefin B., Theopold U. (2014). Damage signals in the insect immune response. Front. Plant Sci..

[85] Soderhall K., Cerenius L. (1998). Role of the prophenoloxidase-activating system in invertebrate immunity. Curr. Opin. Immunol..

[86] Nappi A.J., Christensen B.M. (2005). Melanogenesis and associated cytotoxic reactions: Applications to insect innate immunity. Insect Biochem. Mol. Biol..

[87] Friggi-Grelin F., Iche M., Birman S. (2003). Tissue-specific developmental requirements of *Drosophila* tyrosine hydroxylase isoforms. Genetics.

[88] Hufton S.E., Jennings I.G., Cotton R.G.H. (1995). Structure and function of the aromatic amino acid hydroxylases. Biochem. J..

[89] Liu C., Yamamoto K., Cheng T., Kadono-Okuda K., Narukawa J., Liu S., Han Y., Futahashi R., Kidokoro K., Noda H. (2010). Repression of tyrosine hydroxylase is responsible for the sex-linked chocolate mutation of the silkworm, *Bombyx mori*. Proc. Natl. Acad. Sci. USA.

[90] Black B.C., Pentz E.S., Wright T.R.F. (1987). The α methyl dopa hypersensitive gene, l(2)amd, and two adjacent genes in *Drosophila melanogaster*: Physical location and direct effects of *amd* on catecholamine metabolism. Mol. Gen. Genet..

[91] Cole S.H., Carney G.E., McClung C.A., Willard S.S., Taylor B.J., Hirsh J. (2005). Two functional but noncomplementing *Drosophila* tyrosine decarboxylase genes. J. Biol. Chem..

[92] Han Q., Ding H., Robinson H., Christensen B.M., Li J. (2010). Crystal structure and substrate specificity of *Drosophila* 3,4-dihydroxyphenylalanine decarboxylase. PLoS ONE.

[93] Mehere P., Han Q., Christensen B.M., Li J. (2011). Identification and characterization of two arylalkylamine *N*-acetyltransferases in the yellow fever mosquito, *Aedes aegypti*. Insect Biochem. Mol. Biol..

[94] Hiragaki S., Suzuki T., Mohamed A.A., Takeda M. (2015). Structures and functions of insect arylalkylamine *N*-acetyltransferase (iaaNAT); a key enzyme for physiological and behavioral switch in arthropods. Front. Physiol..

[95] Dyda F., Klein D.C., Hickman A.B. (2000). GCN5-Related *N*-acetyltransferases: A structural overview. Annu. Rev. Biophys. Biomol. Struct..

[96] Dai F., Qiao L., Tong X., Cao C., Chen P., Chen J., Lu C., Xiang Z. (2010). Mutations of an arylalkylamine *N*-acetyltransferase, Bm-iaaNAT, are responsible for silkworm melanism mutant. J. Biol. Chem..

[97] Zhan S., Guo Q., Li M., Li J., Miao X., Huang Y. (2010). Distribution of an *N*-acetyltransferase gene in the silkworm reveals a novel role in pigmentation. Development.

[98] Qiao L., Li Y., Xiong G., Liu X., He S., Tong X., Wu S., Hu H., Wang R., Hu H. (2012). Effects of altered catecholamine metabolism on pigmentation and physiological properties of sclerotized regions in the silkworm melanism mutant. PLoS ONE.

[99] Osanai-Futahashi M., Ohde T., Hirata J., Uchino K., Futahashi R., Tamura T., Niimi T., Sezutsu H. (2012). A visible dominant marker for insect transgenesis. Nat. Commun..

[100] Richardt A., Kemme T., Wagner S., Schwarzer D., Marahiel M.A., Hovemann B.T. (2003). Ebony, a novel nonribosomal peptide synthetase for β-alanine conjugation with biogenic amines in *Drosophila*. J. Biol. Chem..

[101] Wittkopp P.J., True J.R., Carroll S. (2002). Reciprocal function of the *Drosophila yellow* and *ebony* proteins in the development and evolution of pigment patterns. Development.

[102] True J.R., Yeh S.D., Hovemann B.T., Kemme T., Meinertzhagen I.A., Edwards T.N., Liou S.R., Han Q., Li J. (2005). *Drosophila* tan encodes a novel hydrolase required in pigmentation and vision. PLoS Genet..

[103] Aust S., Brusselbach F., Putz S., Hovemann B.T. (2010). Alternative tasks of *Drosophila* tan in neurotransmitter recycling versus cuticle sclerotization disclosed by kinetic studies. J. Biol. Chem..

[104] Philips A.M., Smart R., Strauss R., Brembs B., Kelly L.E. (2005). The Drosophila *black* enigma: The molecular and behavioral characterization of the *black* mutant allele. Gene.

[105] Arakane Y., Lomakin J., Beeman R.W., Muthukrishnan S., Gehrke S., Kanost M.R., Kramer K.J. (2009). Molecular and functional analyses of amino acid decarboxylases involved in cuticle tanning in *Tribolium castaneum*. J. Biol. Chem..

[106] Dai F., Qiao L., Cao C., Liu X., Tong X., He S., Hu H., Zhang L., Wu S., Tan D. (2015). Aspartate decarboxylase is required for a normal pupa pigmentation pattern in the silkworm, *Bombyx mori*. Sci. Rep..

[107] Jones S.M., Solomon E.I. (2015). Electron transfer and reaction mechanism of laccases. Cell. Mol. Life Sci..

[108] Dittmer N.T., Kanost M.R. (2010). Insect multicopper oxidase: Diversity, properties and physiological roles. Insect Biochem. Mol. Biol..

[109] Zhukhlistova N.E., Zhukova Y.N., Lyashenko A.V., Zaitsev V.N., Mikhailov A.M. (2008). Three dimensional organization of three-domain copper oxidases: A review. Crystallogr. Rep..

[110] Dittmer N.T., Gorman M.J., Kanost M.R. (2009). Characterization of endogenous and recombinant forms of laccase-2, a multicopper oxidase from the tobacco hornworm, *Manduca sexta*. Insect Biochem. Mol. Biol..

[111] Futahashi R., Tanaka K., Matsuura Y., Tanahashi M., Kikuchi Y., Fukatsu T. (2011). Laccase 2 is required for cuticular pigmentation in stinkbugs. Insect Biochem. Mol. Biol..

[112] Gorman M.J., Sullivan L.I., Nguyen T.D., Dai H., Arakane Y., Dittmer N.T., Syed L.U., Li J., Hua D.H., Kanost M.R. (2012). Kinetic properties of alternatively spliced isoforms of laccase-2 from *Tribolium casteneum* and *Anopheles gambiae*. Insect Biochem. Mol. Biol..

[113] Yatsu J., Asano T. (2009). Cuticle laccase of the silkworm, *Bombyx mori*: Purification, gene identification and presence of its inactive precursor in the cuticle. Insect Biochem. Mol. Biol..

[114] Paskewitz S.M., Andreev O. (2008). Silencing the genes for dopa decarboxylase or dopachrome conversion enzyme reduces melanization of foreign targets in *Anopheles gambiae*. Comp. Biochem. Physiol. B Biochem. Mol. Biol..

[115] Huang C.Y., Christensen B.M., Li J. (2005). Role of dopachrome isomerase conversion enzyme in the melanization of filarial worms in mosquitoes. Insect Mol. Biol..

[116] De Gregorio E., Spellman P.T., Rubin G.M., Lemaitre B. (2001). Genome-wide analysis of the *Drosophila* immune response by using oligonucleotide microarrays. Proc. Natl. Acad. Sci. USA.

[117] Dong Y., Aguilar R., Xi Z., Warr E., Mongin E., Dimopoulos G. (2006). *Anopheles gambiae* immune responses to human and rodent plasmodium parasite species. PLoS Pathol..

[118] Drapeau M.D. (2001). The family of yellow-related *Drosophila melanogaster* proteins. Biochem. Biophys. Res. Commun..

[119] Walter M.F., Black B.C., Afshar G., Kermabon A., Wright T.R.F., Biessmann H. (1991). Temporal and spatial expression of the *yellow* gene in correlation with cuticle formation and dopa decarboxylase activity in *Drosophila* development. Dev. Biol..

[120] Wittkopp P.J., Vaccaro K., Carroll S. (2002). Evolution of *yellow* gene regulation and pigmentation in *Drosophila*. Curr. Biol..

[121] Wittkopp P.J., Carroll S., Kopp A. (2003). Evolution of black and white: Genetic control of pigment patterns in *Drosophila*. Trends Genet..

[122] Drapeau M.D. (2003). A novel hypothesis on the biochemical role of the *Drosophila* yellow protein. Biochem. Biophys. Res. Commun..

[123] Slominski A., Tobin D.J., Shibahara S., Wortsman J. (2004). Melanin pigmentation in mammalian skin and its hormonal regulation. Physiol. Rev..

[124] Slominski A. (2009). Neuroendocrine activity of the melanocyte. Exp. Dermatol..

[125] D’Mello S.A.N., Finlay G.J., Baguley B.C., Askarian-Amiri M.E. (2016). Signaling pathways in melanogenesis. Int. J. Mol. Sci..

[126] Slominski A., Zmijewski M., Pawelek J. (2012). l-tyrosine and l-dopa as hormone-like regulators of melanocytes functions. Pigment Cell Melanoma Res..

